# Differential Life Satisfaction in a German Representative Sample

**DOI:** 10.3390/ijerph22010105

**Published:** 2025-01-14

**Authors:** Christopher Arnold, Beate Muschalla

**Affiliations:** Department of Psychotherapy and Diagnostics, Technische Universität Braunschweig, 38106 Braunschweig, Germany; b.muschalla@tu-braunschweig.de

**Keywords:** stress, life satisfaction, subjective well-being, resilience, quality of life

## Abstract

Life satisfaction includes various aspects, such as satisfaction with work, family, environment, and finances, and is influenced by sociodemographic and socioeconomic factors. This representative study investigates differential life satisfaction in the general population and its associations with sociodemographics. The study used a cross-sectional design with 2522 German participants, collected via face-to-face interviews and three-stage random sampling, assessing satisfaction across 17 life areas with the Differential Life Burden Scale. Overall life satisfaction was high (*M* = 4.46, scale from 1 to 6). Although globally satisfied, most participants (84.2%) named at least one negative area of life. Politics and environment were perceived as dissatisfying (*M* = 3.1; *M* = 3.81, respectively); social contacts and leisure time were evaluated as rather satisfying (*M* about 5.00). Age, income, and unemployment were associated with life satisfaction. Gender and age were differently associated with life domains: Older people were less satisfied with their health. Younger people were more satisfied with leisure time. Younger were less satisfied with their children than older participants. The German population is generally satisfied with life, though factors like age, unemployment, and income influence the number of negatively perceived life domains. This highlights the importance of evaluating specific life areas in addition to overall life satisfaction for a more comprehensive understanding.

## 1. Introduction

Critical life events and psychological burdens are a part of life. They are general challenges that everyone has to overcome. Everyday stressful situations and psychological burdens are not the only common situations people experience repeatedly. Even less common critical life events are not unusual and are experienced at one point or another in life [1,2,3]. Whether people react “stressed” when confronted with a problem depends on many factors. One of the decisive factors is the resources the individual has to cope with the problem [4,5,6].

A wide variety of person and situation characteristics have emerged in recent decades that are said to have an influence on life stress and, as a counterpart, life satisfaction. Mainly, characteristics such as employment, gender, age, income, and personality have often been examined in recent decades [7,8,9,10,11,12,13]. Besides individual characteristics, environmental and socialization aspects, such as cultural and religious context, may impact life satisfaction [14,15]. The influences of the various aspects are not always clear-cut.

The extent of gender differences in life satisfaction has been investigated many times. One large-scale meta-analysis by Batz-Barbarich et al. [16] compiled over one million study participants. Overall, no gender differences were found with regard to either life satisfaction or job satisfaction [16]. In case significant differences are found between women and men, the effects are rather inconsistent or small [8,17,18,19,20]. While different trends can be identified that point in one direction or another, a number of other influencing factors (personality, region, age, etc.) have been identified in the literature that seem to overshadow the pure effect of gender. It is, therefore, questionable whether gender is a major influencing factor.

Age seems to play a role as well, but also rather specific: Younger women report higher life satisfaction than younger men. However, older men report higher life satisfaction than older women [8]. Similarly, the life satisfaction of adolescents has been found to be lower than that of children [21,22]. Many studies assume a U-shaped progression of life satisfaction over the age span [9,23,24]. While life satisfaction decreases in middle age, it increases again around the age of 50 [23]. However, the studies appear to be problematic in terms of generalizability.

Besides basic sociodemographic characteristics, age and gender, some external aspects should be considered when discussing life satisfaction: Life satisfaction can be associated with a person’s economic situation, i.e., in terms of income. Research showed a positive correlation between income and life satisfaction [25,26,27]. It is important to note that the effect of relative income has been emphasized in the past as it is today. It is less important how high the absolute income is, but rather how high the income is in comparison to the respective environment [25,27,28]. Income insecurity can be a direct stressor and is associated with lower life satisfaction [29], but this relationship is also moderated by personality aspects [13,30].

Besides income, another economic aspect can be important for life satisfaction, i.e., the employment situation: unemployment comes along with reduced life satisfaction [7,31,32]. McKee-Ryan et al. [33] made a major contribution with their meta-analysis. It was found that unemployment generally has a negative impact on life satisfaction. A number of secondary factors, such as financial worries and work role centrality, are suspected. In addition, secondary stressors such as worry, uncertainty, and financial, family, and marital problems also play a role. The duration of unemployment, especially long-term unemployment, as well as the current life status, i.e., whether the person has already worked for many years or became unemployed directly after leaving school, appears to influence the relationship between unemployment and life satisfaction [33,34].

Next to these external factors, life satisfaction is influenced by various thinkable individual differences in cognition and personality: Epictetus already discussed people’s cognitive evaluation of life situations in his “Enchiridion” around 2000 years ago. “Men are disturbed, not by things, but by the principles and notions which they form concerning things.” [35] (p. 17). Beginning in the 20th century, more recent research also addressed the topic. According to Lazarus’ transactional stress theory [36], whether stress, distress or no stress perception results after a stressor depends on the person and the environment. Therefore, life domains can be evaluated by the person as a burden or as a resource. Methods of cognitive reframing can be used to improve life satisfaction perception [6,37].

It should be noted that some authors have not only found differences in global life satisfaction but also in different areas of life [38,39,40,41]. The fact that different areas of life are not rated equally positively by different groups may be due, among other reasons, to the different relevance of the areas of life [39]. This shows that in addition to global life satisfaction, differential life satisfaction is also relevant, which is why instruments that take different areas of life such as family, job, health and environment satisfaction into consideration have been published for this purpose [42].

The literature suggests that stress and life satisfaction perception depend very much on the individual and his/her pattern of sociodemographic, personality and environmental characteristics. Similar stressors are assessed differently by different people (according to Lazarus [36]). There are some core characteristics, i.e., age, income, and unemployment, which might influence how stressors are perceived by many persons more generally. Findings on life satisfaction correlates are partly clear but partly contradictory. Not much is known about life satisfaction concerning specific life domains. For this reason, we investigate descriptively how different areas of life are evaluated in a representative sample of the German population.

The research questions are the following:

1. In which way are gender, age, income and employment situation associated with life satisfaction?
2. Are there differences between men and women in their global and domain-specific life satisfaction?
3. Are there differences between age groups in their global and domain-specific life satisfaction?

## 2. Materials and Methods

The cross-sectional data reported here was collected between March and May 2022. The sample consists of 2522 people from Germany. Face-to-face interviews were conducted with randomly selected people from the general population. The survey was administered by a professional social research company (USUMA GmbH Berlin). The company has many years of experience in the field of population-representative surveys. The sample was drawn using a three-stage process. In the first step, the sample areas were selected by random sampling. The social research company works with a total of 250 sample areas throughout Germany. Ten interviews were conducted in each sample area. In the second step, households in the sample areas were selected at random. This was conducted with the help of an on-site inspection. The third and final step was the selection of the interviewee. The interviewer identified all households and then randomly selected an interviewee. Only one person per household was interviewed. For the last stage, the Kish selection grid method [43] was used. The study was approved by the ethics committee of the University Leipzig prior to any data collection.

The interviewers conducting the interviews were specially trained for the scheduled interviews and received fixed work instructions. A total of 193 interviewers conducted the interviews. This results in an average of around 13 interviews per interviewer. Informed consent was obtained from the interviewees in advance.

The interviews were conducted in person on-site. In addition to the assessment of life satisfaction in different life domains (with the DLB scale) [42], sociodemographic data were recorded (date of birth, gender, marital status, nationality, religion, school-leaving qualification, questions on employment, income, mental health, place of residence).

### 2.1. DLB Scale

The Differential Life Burden Scale (DLB Scale) [42] is a self-assessment questionnaire for negative and positive emotions in relation to various areas of life. The questionnaire consists of 17 items in the areas of partnership/marriage, sexuality, children, parents, friends, neighbors/acquaintances, colleagues, work, leisure, health, finances, residence, environment, homeland, politics, future, and life balance. The questionnaire begins with: “Below you will find a list of important areas of life. Please indicate with a cross which of the following answers most closely matches your feelings when you think of the corresponding area of life. Please do not skip a single line!”. This is followed by the 17 areas of life. Answers can be given on a six-point scale with the levels “very negative”, “negative”, “slightly negative”, “slightly positive”, “positive”, and “very positive”. According to the authors [42], the completion time is very short at approximately 3 min. The DLB scale has good internal consistency (Cronbach’s alpha = 0.74–0.77) [42]. According to the authors, the calculation of the item intercorrelation showed low to medium-high correlations between the items.

### 2.2. Statistics

The statistical analyses were performed using IBM SPSS Statistics© (version 27.0.0.0; 64-bit version). All statistical analyses were based on an alpha level of 0.05.

## 3. Results

Initially, a sample description is provided to facilitate an assessment of its representativeness. A total of *N* = 2522 study participants were surveyed. Every person in Germany over the age of 15 was eligible to participate in the study. The age of all test subjects was between 16 and 96 years and averaged 49.26 years (*SD* = 17.655). The gender was 49.7% male, 50.1% female, and 0.2% diverse. A total of 96.4% stated that they had German citizenship. Further demographic data can be found in Table 1.

On average, the investigated participants had already been unemployed 1.02 times in their lives. Currently unemployed were 4% of the sample. Further work-related data and data on income can be found in Table 2 and Table 3, respectively.

### 3.1. Domains of Life with Comparably Low Satisfaction

The frequencies of negative responses show which are the life dimensions with low satisfaction: A minority of people (15.8%) rated none of the listed life domains to any degree negative (“slightly negative”, “negative”, “very negative”). Half of the sample (51.3%) rated 3 or more life domains in any way negative.

However, excluding “slightly negative”, 48.5% rated none of the listed life areas as “negative” or “very negative”. Lastly, 79.6% of participants rated no life domain “very negative”.

### 3.2. Global Life Satisfaction

Gender, age, income, and current unemployment may be associated with global life satisfaction, both as a stressor and as a resilience factor. These sociodemographics are included in a regression model in order to investigate their influence on life satisfaction: “gender” (male, female), “age” (in years), “income” (low, high), and “currently unemployed” (yes, no). For the variable “gender”, “diverse” was excluded from the study, as the subgroup would have been too small with only four participants.

Results from the regression analysis are reported in Table 4. With a corrected R^2^ = 0.074, the model has a low goodness-of-fit [44]. The predictors “age”, “income”, and “current unemployment” significantly explained the variance of the criterion “number of negatively rated areas of life”.

### 3.3. Differential Measurement of Life Satisfaction

The empirical literature indicates that life satisfaction might be influenced by gender and age, not on a global level but on a differential level. For this reason, we examined whether or not there are differences in differential life satisfaction between women and men (Table 5) and between different age groups (Table 6).

Men and women reported satisfaction with most life domains on a similar level. Gender differences were observed in only 3 out of 17 life domains (Table 5). All differences were found for domains associated with the nuclear family: In “partnership” and “sexuality”, male participants reported higher satisfaction levels compared to females. Women were more satisfied with “children” compared to men.

The comparison across age groups revealed significant differences in almost all (15 out of 17) life domains. The direction of these differences varied by the domain (Table 6): Younger and middle-aged people reported that they were more satisfied with work-related areas of life (i.e., “colleagues”, “work”), close acquaintances and family (i.e., “parents”, “friends”) as well as sexuality, health, and their future. In middle to old age, compared to younger people, there is a greater satisfaction level with monetary and materialistic areas of life (i.e., “finances”, “residence”), society-related areas (i.e., “homeland”, “politics”) and one’s own children. With regard to the environment and life balance, a similar assessment was found across all age groups.

## 4. Discussion

This is one of the first representative studies on the distribution and basic characteristics of differential life satisfaction in a general population. Gender ratio, age, marital status, and educational level largely correspond with the characteristics of the population [45,46,47,48,49]. The presented data can be interpreted as representative data.

### 4.1. General Life Satisfaction in the General Population

First of all, it should be noted that the representative sample is above average in 16 of 17 life satisfaction areas. The life domains of friends, leisure, homeland, and parents were rated as the four most positive life domains. Habich [50] found similar results even 30 years ago. He examined various areas of life (marriage/partnership, leisure, homeland, etc.) and found that the majority of the German population was satisfied in many areas of life, with satisfaction levels of over 90% in some cases. Globally, most people in Germany are more satisfied than dissatisfied [32,51]. Even despite the negative impact of the pandemic, people in Germany were, on average, satisfied at this time [51].

In contrast, politics and the environment were rated as the most negative areas of life. Politics was the only area of life below the scale mean of 3.5 points and was therefore rated more negatively than positively. This is in line with other research findings: In the study by Habich [50], politics and the environment, together with the area of “public safety”, are also the worst-rated domains of life. The environment also received one of the lowest ratings in Priem et al. [52]. The negative perception of politics fits to present naturalistic investigations: In a survey of 1321 eligible voters in Germany, around half (46%) answered to be “not at all satisfied” to the question “How satisfied are you with the work of the federal government?”. A further 36% were “less satisfied”. Only 16% were “satisfied” and only 1% of participants were “very satisfied” [53]. Thus, most eligible voters are dissatisfied with politics.

Why are people dissatisfied with life domains that are rather “far away” and do not even touch their own day-to-day life (e.g., reports about war in other countries)? An explanation can be cognitively mediated emotions induced by the media reports. Most people in Germany use a wide variety of media on a daily basis [54,55,56]. News is often about problems (climate change, war), presented with emotionality and negative bias. Thus, being dissatisfied with politics and the larger environment could therefore be interpreted as a case of “cognitively mediated suffering”: Without a direct experience of personal suffering (there is no war in one’s own town), a negative attitude is nevertheless developed simply by negatively framed information.

Despite being overall rather satisfied with most life domains, almost all respondents, i.e., around 84.2%, rated at least one area of life as “slightly negative”, “negative”, or “very negative”. The data shows that negative areas of life occur frequently in the general population. Negative events are normal [1,2,3]. It would be rather unusual for someone to have no negative life domain at all.

While it was common for several life domains to be rated “negative” or “slightly negative”, it was exceptional for several life domains to be rated as “very negative”. This is in line with previously published results: McGinty et al. [3] report an average of around 3 to 4 critical life events from people in different countries. Critical life events can occur at any age and in any other group of population subdivisions. These critical life events can be the temporal onset of maladaptive processes and are thus related to the prevalence figures of this study [3]. Similarly, the prevalence figures can also be linked to the results of Hyland et al. [1]. Critical life events, maladaptive processes, and the resulting negative evaluations are not uncommon and are experienced by a large proportion of the population at some point in their lives. However, they are regularly demanding but normal and surmountable (e.g., illness of a family member).

Our findings on a globally satisfied population, with differences in satisfaction in different life domains, fit the results of previous studies on global life satisfaction [7,31,32,57,58,59,60], as well as the domain-specific studies [50,52,61].

### 4.2. General Life Satisfaction and Sociodemographics

The data from this representative study confirm earlier findings [16,62]. There was no relevant relation between gender and overall life satisfaction, according to the other literature: Gender is not predictive of global life stress [17,18,19,20].

Also, age was rather independent of life satisfaction. Some research results also indicate no correlation between age and life satisfaction [22,63]; some results show a positive correlation [64]. However, life satisfaction and age might not be linearly correlated; there could also be a U-shaped relationship between life satisfaction and age [9,23,24].

Life satisfaction seems to have a positive correlation with income [25,27,28,65]. In the here investigated representative sample, a higher income was associated with fewer negatively rated life domains compared to people with a lower income. However, there may also be a moderator effect of personality here [13,30]. It is unclear to what extent this influence would have changed if relative income would have been examined [25,27,28].

Current unemployment had the greatest association with life dissatisfaction. This corresponds with the current literature [7,31,32,33].

### 4.3. Specific Life Satisfaction in Different Life Domains

“Partnership/marriage” and “Sexuality” were rated significantly more stressful by women than by men. The area of “children” was rather rated worse by men.

The two areas that were rated significantly worse by women than by men (“partnership/marriage” and “sexuality”) are both areas that are directly related to the partnership. This is in contrast to the other life areas, where women are at least as satisfied as men. Similar results were found for married couples in the meta-analysis by Jackson et al. [66]. The results of our data and the literature suggest that women are more dissatisfied with their own partnership compared to men.

With regard to satisfaction with one’s children, the literature is partly divided. While some studies found no gender differences [67], there are also reports of higher stress experienced by mothers [41,68]. Stress levels may be dependent on external conditions, e.g., there was somewhat higher stress perception in parents during a pandemic (which came along with restrictions such as school closing and homeschooling necessities additional to normal daily duties [40]).

There were significant differences in satisfaction in nearly all life domains between different age groups. Only “environment” and “life balance” were perceived with similar satisfaction over all age groups. The life area “leisure” was rated significantly more satisfying by young people than by middle-aged or older people. The satisfaction with “children” was the opposite: young respondents were significantly less satisfied with their children than middle-aged or older people.

One possible explanation for different levels of satisfaction with different life domains in different age groups is the different relevance of the subject domains [39]. Our data fit this assumption: while “work” is highly relevant in young and middle age, it may no longer be as relevant in older age. On the other hand, satisfaction with “health” was rated significantly lower in the older age group. Illnesses and a reduction in health may occur as a stressor in old age rather than in young age [69].

### 4.4. Limitations and Future Research

Although the sample is large and representative of the German general population, it is limited in that no statements can be made about children and adolescents younger than 16 years. It can be assumed that life domains are weighted differently in childhood and adolescence.

Furthermore, not all thinkable aspects that might explain variance in life satisfaction have been assessed. Other variables influencing life satisfaction should be examined in further studies, e.g., personality, which is repeatedly regarded in the literature.

## 5. Conclusions

The data show that the German population is generally satisfied in most life domains. While it is not uncommon to feel slightly negative about some life domains, it was rare to have several very negative life domains. Only very few participants reported more negative than positive life domains.

(Un)employment, income, and age (but not gender) have a relevant impact on global or domain-specific life satisfaction.

The study underlines that global life satisfaction does not capture the overall picture. Differential life satisfaction should be considered in clinical or counseling practice and research by asking for satisfaction in different life domains.

## Figures and Tables

**Table 1:** Sample characteristics of the representative German national sample (*N* = 2522).

Variable	Frequency in %		
Age Groups	Female	Male	All
Under 20	3.9	3	3.4
20–40	27.7	30.3	29.1
40–60	38.1	36	37.0
60–80	25.8	26.1	25.9
80–100	4.5	4.6	4.6
Graduation			
Without a lower secondary school qualification	2.3	2.5	2.4
Lower secondary school qualification	23.9	25.4	24.6
Secondary school qualification	37.2	29.8	33.5
Polytechnic secondary school	8.2	8.7	8.4
Technical school qualification (without recog. as univ. of appl. sciences)	3.7	5.1	4.4
General or subject-specific higher education entrance qualification	12.6	13.7	13.1
Completed university/college degree	9.6	13.5	11.6
Other qualification	0.4	0.1	0.2
Pupil at a general education school	2.1	1.2	1.7
Marital status			
Married (living together)	42.3	50.3	46.2
Married (living separately)	1.5	1.8	1.6
Unmarried	26.5	31.8	29.2
Divorced	17.9	10.8	14.4
Widowed	11.8	5.3	8.5

**Table 2:** Work status of the representative German national sample (N = 2522).

Variable	Frequency in %		
Employment	Female	Male	All
Full-time employment 35 h or more	34.8	62.8	48.7
Part-time employed 15–34 h	20.9	3.2	12.1
Employed hourly	4.4	0.5	2.4
Voluntary service/maternity leave	1.5	0.1	0.8
Currently unemployed	3.5	4.4	4.0
Pensioner/retired/in early retirement	24.8	23.6	24.2
Not working, e.g., housewife/househusband	3.8	0.6	2.2
In vocational training	0.8	1.6	1.2
In-school education (also university, college)	5.5	3.2	4.3
Unemployment (Group)			
Never	51.9	56.1	53.8
Once	20	19.1	19.5
Multiple times	28.1	24.8	26.4
Employment (Group)			
Employed	60.1	66.5	63.3
In school vocational training/voluntary service	7.8	4.9	6.3
Currently unemployed	3.5	4.4	4.0
Pensioner/retired/in early retirement	24.8	23.6	24.2
Not working: e.g., housewife/househusband	3.8	0.6	2.2
Profession			
Never been employed	6.6	4.3	5.5
Self-employed without employees	2	2	2.0
Self-employed with up to 5 employees	1.1	2.9	2.0
Self-employed with 6–9 employees	0.2	0.5	0.3
Self-employed with 10–49 employees	0.4	0.5	0.4
Self-employed with 50 or more employees	0.2	0	0.1
Independent profession	1	1.7	1.3
Simple employees, tasks without authority to issue instructions	30	6	18.1
Intermediate employees	24.6	13.2	19.0
Qualified employees	20.4	21.6	21.0
Senior executives with signing authority	2	4.7	3.3
Public official–elementary service	0.6	0.5	0.5
Public official–intermediate service	1.4	2.6	2.1
Public official–higher service	1.3	2.5	1.9
Public official–senior service	0.2	0.7	0.5
Laborers simple work (mainly physical strength)	4	6.3	5.1
Laborer difficult work (mainly skill)	1.7	5.4	3.5
Skilled worker, foreman, journeyman	2.2	24.4	13.2
Self-employed farmers	0.1	0.2	0.2

**Table 3:** Income of the representative German national sample (N = 2522).

Variable	Frequency in %		
Personal income	Female	Male	All
No personal income	6.2	3.1	4.6
<500 €	4.5	1.1	2.8
500 -< 650 €	3.7	1.3	2.5
650 -< 750 €	3.1	0.7	1.9
750 -< 900 €	7	3.1	5.1
900 -< 1000 €	6	2.1	4.0
1000 -< 1150 €	7.2	3.9	5.6
1150 -< 1250 €	7.5	3.7	5.6
1250 -< 1500 €	15.4	10.4	12.9
1500 -< 2000 €	19.5	23.9	21.7
2000 -< 2500 €	12	23	17.5
2500 -< 3500 €	5.6	16	10.8
3500 -< 5000 €	1.7	6.4	4
≥5000 €	0.6	1.3	0.9
Household income			
<1250 € per month	10.1	8.2	9.1
≥1250 -< 2500 € per month	42.1	35.6	38.9
≥2500 € per month	47.8	56.2	52.0

**Table 4:** Multiple linear regression-prediction of the number of negative areas of life.

Coefficients	*b*	SE	*β*	*t*	*p*	95% CI	
						*LB*	*UB*
(Constant)	2.582	0.280		9.205	<0.001	2.032	3.132
Gender	0.109	0.13	0.017	0.803	0.422	−0.15	0.358
Age	0.013	0.004	0.073	3.596	<0.001	0.006	0.02
Income	−1.173	0.177	−0.139	−6.646	<0.001	−1.519	−0.827
Currently unemployed	3.459	0.331	0.214	10.452	<0.001	2.81	4.108

Note. N = 2246; R^2^ = 0.074; corr. R^2^ = 0.073; *F*(*4, 2246*) = 45.058, *p* < 0.001.

**Table 5:** Satisfaction with different domains of life of the representative German national sample (N = 2522).

Variable	Male	Female	All	Sig.
	*M* (*SD*)	*M* (*SD*)	*M* (*SD*)	
Partnership/Marriage	4.64 (1.36)	4.46 (1.49)	4.55 (1.43)	*F*(*1*, *2482*) = 10.189, *p* = 0.001
Sexuality	4.53 (1.31)	4.23 (1.42)	4.38 (1.37)	*F*(*1*, *2472*) = 29.965, *p* = <0.001
Children	4.6 (1.35)	4.81 (1.31)	4.71 (1.33)	*F*(*1*, *2415*) = 14.702, *p* = <0.001
Parents	4.75 (1.14)	4.79 (1.15)	4.77 (1.14)	*F*(*1*, *2457*) = 0.686, *p* = *n.s.*
Friends	5.05 (0.81)	5.06 (0.83)	5.06 (0.82)	*F*(*1*, *2506*) = 0.033, *p* = *n.s.*
Neighbors/acquaintances	4.47 (0.94)	4.47 (1)	4.47 (0.97)	*F*(*1*, *2506*) = 0.022, *p* = *n.s.*
Colleagues	4.5 (0.95)	4.52 (1.01)	4.51 (0.98)	*F*(*1*, *2506*) = 0.337, *p* = *n.s.*
Work	4.5 (1.09)	4.48 (1.13)	4.49 (1.11)	*F*(*1*, *2440*) = 0.207, *p* = *n.s.*
Leisure	5 (0.84)	4.97 (0.88)	4.99 (0.86)	*F*(*1*, *2507*) = 1.075, *p* = *n.s.*
Health	4.68 (1.09)	4.6 (1.15)	4.64 (1.12)	*F*(*1*, *2505*) = 3.793, *p* = *n.s.*
Finances	4.26 (1.19)	4.17 (1.22)	4.21 (1.21)	*F*(*1*, *2505*) = 3.922, *p* = *n.s.*
Residence	4.76 (1)	4.8 (1.03)	4.78 (1.02)	*F*(*1*, *2508*) = 1.213, *p* = *n.s.*
Environment	3.84 (1.15)	3.79 (1.19)	3.81 (1.17)	*F*(*1*, *2506*) = 1.306, *p* = *n.s.*
Homeland	4.65 (.99)	4.66 (1.04)	4.65 (1.02)	*F*(*1*, *2504*) = 0.096, *p* = *n.s.*
Politics	3.15 (1.18)	3.05 (1.19)	3.1 (1.19)	*F*(*1*, *2497*) = 4.689, *p* = *n.s.*
Future	4.01 (1.18)	3.96 (1.22)	3.99 (1.2)	*F*(*1*, *2507*) = 1.268, *p* = *n.s.*
Life balance	4.5 (1.01)	4.46 (1.06)	4.48 (1.03)	*F*(*1*, *2508*) = 0.651, *p* = *n.s.*

Note. *M*: mean value; *SD*: standard deviation; *n.s.* = not significant.

**Table 6:** Satisfaction with different domains of life in different age groups (N = 2522).

Variable	Young (≤30 Years Old) (*N* = 456)	Middle (31–60 Years Old) (*N* = 1341)	Old (>60 Years Old) (*N* = 725)	Sig.	Post-Hoc
	*M* (*SD*)	*M* (*SD*)	*M* (*SD*)		
Partnership/Marriage	4.53 (1.38)	4.65 (1.41)	4.38 (1.48)	*F*(*2*, *2485*) = 8.627, *p* = <0.001	M vs. *O* (*p* < 0.001, *M*_Diff_ = 0.274, 95%-CI [0.12, 0.43])
Sexuality	4.7 (1.28)	4.59 (1.28)	3.79 (1.42)	*F*(*2*, *2475*) = 98.299, *p* = <0.001	Y vs. O (*p* < 0.001, (*M*_Diff_ = 0.905, 95%-CI [0.71, 1.1]) M vs. O (*p* < 0.001, *M*_Diff_ = 0.793, 95%-CI [0.64, 0.94])
Children	4.18 (1.52)	4.79 (1.3)	4.85 (1.19)	*F*(*2*, *2417*) = 40.996, *p* = <0.001	Y vs. M (*p* < 0.001, *M*_Diff_ = −0.617, 95%-CI [−0.79, −0.44]) Y vs. O (*p* < 0.001, *M*_Diff_ = −0.677, 95%-CI [−0.87, −0.48])
Parents	5.02 (1)	4.78 (1.12)	4.57 (1.24)	*F*(*2*, *2459*) = 21.141, *p* = <0.001	Y vs. M (*p* = 0.001, *M*_Diff_ = 0.232, 95%-CI [0.08, 0.38]) Y vs. O (*p* < 0.001, *M*_Diff_ = 0.444, 95%-CI [0.28, 0.61]) M vs. O (*p* < 0.001, *M*_Diff_ = 0.213, 95%-CI [0.08, 0.34])
Friends	5.32 (0.81)	5.04 (0.81)	4.91 (0.8)	*F*(*2*, *2509*) = 37.601, *p* = <0.001	Y vs. M (*p* < 0.001, *M*_Diff_ = 0.28, 95%-CI [0.17, 0.38]) Y vs. O (*p* < 0.001, *M*_Diff_ = 0.417, 95%-CI [0.3, 0.53]) M vs. O (*p* = 0.001, *M*_Diff_ = 0.138, 95%-CI [0.05, 0.23])
Neighbors/acquaintances	4.41 (1)	4.43 (0.97)	4.58 (0.95)	*F*(*2*, *2509*) = 6.48, *p* = 0.002	Y vs. O (*p* = 0.009, *M*_Diff_ = −0.173, 95%-CI [−0.31, −0.03]) M vs. O (*p* = 0.004, *M*_Diff_ = −0.146, 95%-CI [−0.25, −0.04])
Colleagues	4.63 (0.93)	4.56 (0.95)	4.34 (1.05)	*F*(*2*, *2421*) = 14.535, *p* = <0.001	Y vs. O (*p* < 0.001, *M*_Diff_ = 0.285, 95%-CI [0.14, 0.43]) M vs. O (*p* < 0.001, *M*_Diff_ = 0.214, 95%-CI [0.1, 0.33])
Work	4.53 (1.11)	4.58 (1.08)	4.31 (1.15)	*F*(*2*, *2443*) = 13.314, *p* = <0.001	Y vs. O (*p* = 0.003, *M*_Diff_ = 0.223, 95%-CI [0.06, 0.39]) M vs. O (*p* < 0.001, *M*_Diff_ = 0.266, 95%-CI [0.14, 0.39])
Leisure	5.11 (0.87)	4.99 (0.87)	4.9 (0.85)	*F*(*2*, *2510*) = 8.518, *p* = <0.001	Y vs. M (*p* = 0.022, *M*_Diff_ = 0.125, 95%-CI [0.01, 0.24]) Y vs. O (*p* < 0.001, *M*_Diff_ = 0.213, 95%-CI [0.09, 0.34])
Health	5.12 (0.98)	4.73 (1.04)	4.16 (1.18)	*F*(*2*, *2508*) = 122.008, *p* = <0.001	Y vs. M (*p* < 0.001, *M*_Diff_ = 0.385, 95%-CI [0.25, 0.52]) Y vs. O (*p* < 0.001, *M*_Diff_ = 0.957, 95%-CI [0.8, 1.11]) M vs. O (*p* < 0.001, *M*_Diff_ = 0.572, 95%-CI [0.45, 0.69])
Finances	4.04 (1.36)	4.25 (1.2)	4.26 (1.1)	*F*(*2*, *2508*) = 5.931, *p* = 0.003	Y vs. M (*p* = 0.004, *M*_Diff_ = −0.209, 95%-CI [−0.37, −0.05]) Y vs. O (*p* = 0.006, *M*_Diff_ = −0.223, 95%-CI [−0.4, −0.05])
Residence	4.57 (1.16)	4.8 (1.01)	4.88 (0.92)	*F*(*2*, *2511*) = 14.127, *p* = <0.001	Y vs. M (*p* < 0.001, *M*_Diff_ = −0.23, 95%-CI [−0.36, −0.1]) Y vs. O (*p* < 0.001, *M*_Diff_ = −0.318, 95%-CI [−0.46, −0.17])
Environment	3.8 (1.26)	3.78 (1.17)	3.88 (1.11)	*F*(*2*, *2509*) = 1.728, *p* = *n.s.*	
Homeland	4.6 (1.06)	4.61 (1.03)	4.78 (0.95)	*F*(*2*, *2506*) = 7.657, *p* = <0.001	Y vs. O (*p* = 0.01, *M*_Diff_ = −0.178, 95%-CI [−0.32, −0.03]) M vs. O (*p* = 0.001, *M*_Diff_ = −0.174, 95%-CI [−0.29, −0.06])
Politics	3.05 (1.2)	3.05 (1.18)	3.21 (1.18)	*F*(*2*, *2500*) = 4.702, *p* = 0.009	M vs. O (*p =* 0.01, *M*_Diff_ = −0.161, 95%-CI [−0.29, −0.03])
Future	4.3 (1.18)	4.01 (1.22)	3.74 (1.14)	*F*(*2*, *2510*) = 32.045, *p* = <0.001	Y vs. M (*p* < 0.001, *M*_Diff_ = 0.295, 95%-CI [0.14, 0.45]) Y vs. O (*p* < 0.001, *M*_Diff_ = 0.564, 95%-CI [0.39, 0.73]) M vs. O(*p* < 0.001, *M*_Diff_ = 0.269, 95%-CI [0.14, 0.4])
Life balance	4.51 (1.04)	4.48 (1.07)	4.46 (0.96)	*F*(*2*, *2511*) = 0.342, *p* = *n.s.*	

Note. *M*: mean value; *SD*: standard deviation; *n.s.* = not significant.

## Data Availability

Data will be made available on reasonable request by the corresponding author.
